# Psychological Interventions for Individuals With Acquired Brain Injury, Cerebral Palsy, and Spina Bifida: A Scoping Review

**DOI:** 10.3389/fped.2022.782104

**Published:** 2022-03-21

**Authors:** Morgan Jefferies, Taylor Peart, Laure Perrier, Andrea Lauzon, Sarah Munce

**Affiliations:** ^1^Department of Occupational Science and Occupational Therapy, University of Toronto, Toronto, ON, Canada; ^2^University of Toronto Libraries, University of Toronto, Toronto, ON, Canada; ^3^LIFESpan Service, Holland Bloorview Kids Rehabilitation Hospital, Toronto, ON, Canada; ^4^LIFESpan Service, Toronto Rehabilitation Institute-University Health Network, Toronto, ON, Canada; ^5^Lawrence S. Bloomberg Faculty of Nursing, University of Toronto, Toronto, ON, Canada

**Keywords:** psychological intervention, transitions, cerebral palsy, spina bifida, acquired brain injury, scoping review

## Abstract

**Background:**

With current medical advancements, more adolescents with neurodevelopmental disorders are transitioning from child- to adult-centred health care services. Therefore, there is an increasing demand for transitional services to help navigate this transition. Health care transitions can be further complicated by mental health challenges prevalent among individuals with cerebral palsy (CP), spina bifida (SB), and childhood onset acquired brain injury (ABI). Offering evidence-based psychological interventions for these populations may improve overall outcomes during transition period(s) and beyond. The objective of this scoping review is to identify key characteristics of psychological interventions being used to treat the mental health challenges of adolescents and adults with CP, SB, and childhood onset ABI.

**Methods:**

Methodological frameworks by Arksey and O'Malley, and Levac and colleagues were used to explore studies published between 2009 and 2019. Included studies were required to be written in English and report on a psychological intervention(s) administered to individuals at least 12 years of age with a diagnosis of CP, SB, or childhood onset ABI. All study designs were included.

**Results:**

A total of 11 studies were identified. Of these, eight reported psychological interventions for childhood onset ABI, while three reported on CP. No studies reporting on SB were identified. Commonly used interventions included acceptance and commitment therapy (ACT), psychotherapy, and cognitive behavioral therapy (CBT).

**Conclusions:**

There are a limited number of studies investigating psychological interventions for individuals with childhood onset ABI and CP, and none for individuals with SB. Further research into effective psychological interventions for these populations will improve mental health outcomes and transitional services.

## Introduction

Neurodevelopmental disorders, including cerebral palsy (CP), spina bifida (SB), and childhood onset acquired brain injury (ABI) are complex medical conditions that may impact multiple aspects of a child's life, including his/her physical and psychological wellbeing (1, 2). Individuals with these conditions often require life-long health care management to reach occupational goals and their fullest potential (3, 4). In the past, children with neurodevelopmental disorders were often not expected to survive into adulthood; however, with current medical advancements, more than 75% will live to adulthood (2, 3, 5). This increase in longevity has subsequently led to an increase in the number of children transitioning from child- to adult-centered health care (6).

The transition from child- to adult-centered health care services is a complex and difficult process (7–9). Numerous studies have reported many transitional services are not coordinated to meet both physical and psychosocial needs (5, 10–13). Indeed, the transition process can further be complicated by psychological difficulties individuals with CP, SB, and childhood onset ABI may experience. Mental health difficulties such as anxiety, depression, and lower self-esteem have been found to be more common among young adults with neurodevelopmental disorders (10, 14, 15). For example, individuals with CP are more likely to experience poorer mental health compared to those without CP (16, 17). It has also been reported that 41–48% of young adults (14–21) with a diagnosis of SB experience depression compared to 10.9% of their typically developing peers (18). Furthermore, individuals with mild to severe traumatic brain injury (TBI) are at a significantly higher risk of developing psychiatric disorders, such as anxiety or depression, compared to their same aged peers (19). Adults with childhood onset conditions are also more likely to experience a lack of community involvement, decreased social skills, peer rejection, and social stigma in comparison to others (2, 20, 21). These psychosocial difficulties can further complicate one's experience of navigating the health care system and managing chronic health conditions.

Psychological interventions may be one option to address these significant concerns (22, 23). Commonly used effective psychological interventions for the general population include cognitive behavioral therapy (CBT) (24, 25), psychotherapy (25), and mindfulness or other relaxation techniques (26, 27). Although many studies have been published investigating the effectiveness of psychological interventions for the general population, literature summarizing psychological interventions and its effectiveness for individuals with neurodevelopmental disorders is lacking (22, 23) (i.e., lack of recognition of the “emotional life” of adults with neurodevelopmental disorders), despite the population's increasing psychological difficulties.

Further exploration of the various psychological interventions for individuals with CP, SB, and childhood onset ABI is essential to improve transitional health care services. By examining the extent of published literature on psychological interventions, gaps within this area of research will be identified and provide insight into potential treatments for mental illnesses these populations experience (22, 23). Thus, the purpose of this scoping review is to (1) determine what psychological interventions have been reported in the literature/evaluated to treat mental health difficulties experienced by individuals with CP, SB, and childhood onset ABI; and (2) identify the key characteristics of these interventions for these populations and their effectiveness.

## Methods

The current scoping review used the methodological frameworks proposed by Arksey and O'Malley (28) and Levac et al. (29) The Preferred Reporting Items for Systematic Reviews and Meta-Analyses Extension for Scoping Reviews (PRISMA-ScR) (30) was used to inform the processes and reporting of this review.

### Eligibility Criteria

To be eligible for inclusion, studies must have investigated the implementation of psychological interventions with individuals with CP, SB, and childhood onset ABI. Psychological interventions were defined as treatments focused on reducing psychological distress through counseling, support, interaction or instruction, with an aim to increase adaptive behavior (31). Examples of psychological interventions include CBT, mindfulness-based stress reduction, or psychotherapy. These interventions can be delivered face-to-face or online (e.g., video conferencing, social media), and be delivered one-on-one or in a group setting by any health care provider or health care provider trainee (i.e., we excluded those interventions that were delivered by a peer mentor). To ensure literature was relevant to the current health care system, only studies published within the last 10 years (e.g., from January 2009 to July 2019) were included. All study designs were included. Furthermore, only studies published in English were included.

### Search Strategy

The literature search strategy was developed by the research team in collaboration with a Librarian (LP) with expertise in systematic and scoping review methods. The search included medical subject headings and text words related to adults with childhood onset disabilities (e.g., CP, SB, or ABI), and psychological interventions (e.g., CBT or mindfulness). Relevant literature was collected from: MEDLINE, CINAHL, EMBASE, and PsycINFO, to ensure literature was collected from a diverse range of disciplines. Appropriate wildcards were used to account for plurals and variations in spelling. The search strategy for MEDLINE can be found in an additional file (see Supplementary File 1). Reference lists from reviews were hand-searched to ensure literature saturation.

### Study Selection

All study screening and selection occurred in Covidence (covidence.org), an online literature review tool. Titles and abstracts of studies identified were screened (i.e., level one screening). Full-text screening of potentially relevant articles (i.e., level two screening) was completed to determine final article inclusion. Both level one and level two screening were done independently by two reviewers (MJ and TP). Conflicts between reviewers were resolved through discussion to reach consensus.

### Data Abstraction

Data abstracted included authors, year of publication, country of study, recruitment setting, mean age/age range, sample size, type of condition, key intervention characteristics, and the associated outcomes and their impact. Intervention characteristics reported were based on the Template of Intervention Description and Replication (TIDieR) framework (32).

## Results

### Study Selection

The literature search yielded 14,126 records. EMBASE, CINAHL, MEDLINE, and PsycINFO retrieved 2,293, 3,242, 3,092, and 2,199, respectively. Following duplication removal, 10,269 records remained. In level two screening, 241 full-text articles were assessed, with 230 articles being excluded. Reasons for exclusion included wrong interventions (e.g., not psychological interventions), wrong population (e.g., adult onset ABI), wrong outcomes (e.g., not mental health outcomes), wrong study design (e.g., review articles or editorials), and inability to locate full-text articles. For conference abstracts, attempts were made to contact authors for full-texts.

**Figure 1:**
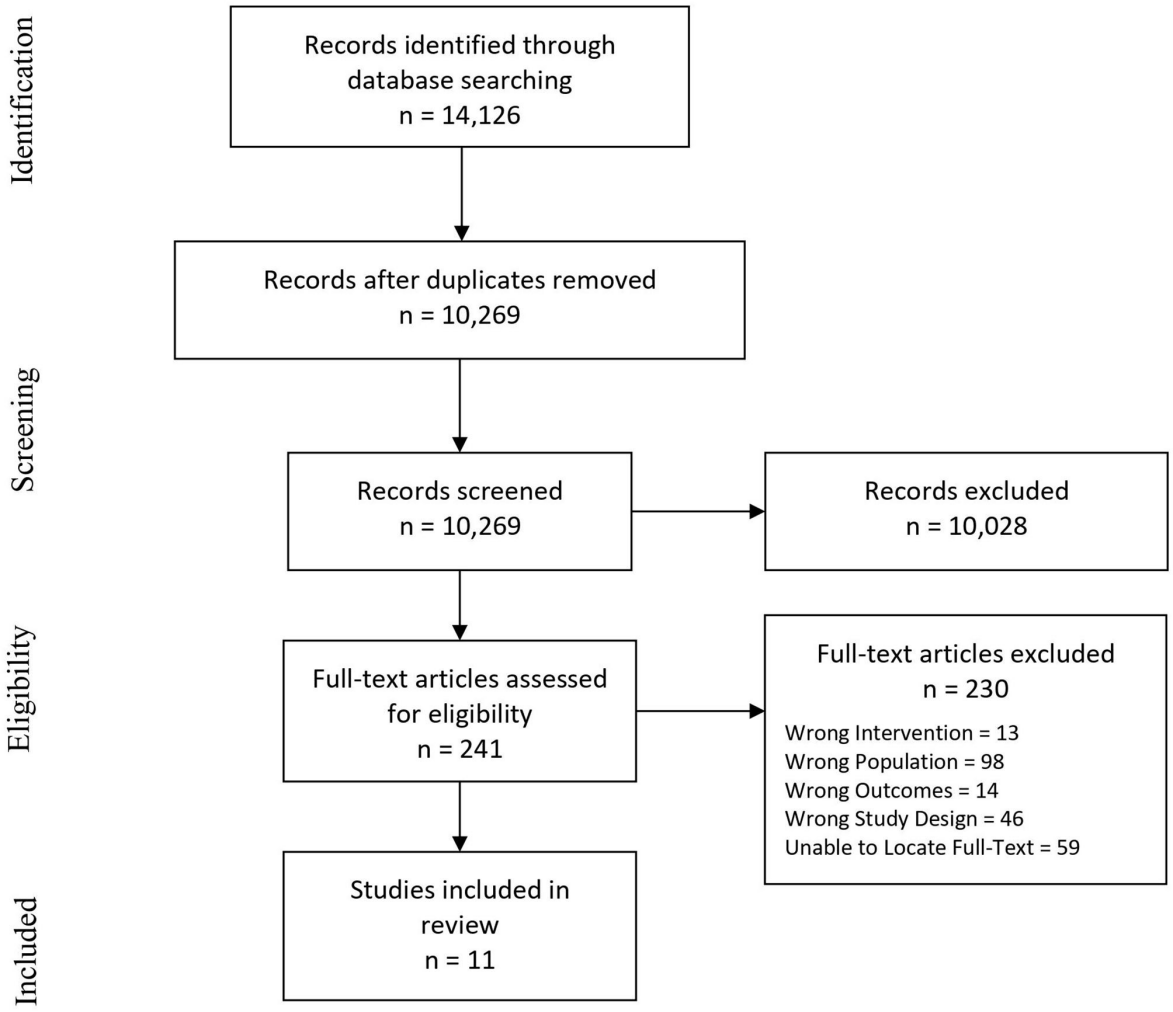
PRISMA flow diagram adapted from Moher, Liberati, Tetzlaff, Altman, & the PRISMA group (2009).

Following screening, 11 studies remained for final inclusion. Studies were summarized with a focus on key intervention characteristics by using the TIDieR framework. Figure 1 outlines the review process using the PRISMA-ScR.

### Identified Studies

Included studies were conducted in the United States (33–37), United Kingdom (38, 39), Australia (40), Greece (41), Poland (42), and Italy (43). Studies were published between 2010 and 2018. Participant recruitment settings included community clinics, hospitals, universities, medical centres, and rehabilitation centres. Study sample sizes ranged from one to 49, with mean ages ranging from 6.87 to 15.9 years. Conditions studied included CP (36, 39, 41) and childhood onset ABI (33–35, 37, 40, 42, 43), consisting of TBI, with only one study having a variety of ABI conditions (40). No studies reported on interventions for SB. Detailed information of the included studies, including the intervention characteristics using the TIDieR framework and effectiveness/impact, can be found in Tables 1–3.

**Table 1:** Summary of psychological interventions using the TIDieR framework items one through six.

**Item study**	**1. Brief name**	**2. Why**	**3. What (materials)**	**4. What (procedure)**	**5.Who provided**	**6.How**
Ashish et al. (34) United States *N* = 1	Multipronged intervention of psychotic symptoms: acceptance and commitment therapy, family therapy	Traumatic brain injury (TBI) is common in children and adolescents. Symptoms following TBI included impaired cognitive functioning and mood disturbances. ACT may help to alleviate mental health problems following TBI. Additionally, multiple systems of therapy have been found to improve outcomes following TBI.	Not specified	Client treatment included: (1) vestibular-ocular physical therapy to promote balance and reduce dizziness; (2) individual therapy which consisted of ACT; (3) family therapy with the patient's family involving role-playing exercises, and encouragement to increase awareness of feelings.	Therapist—nothing further specified	Delivered face-to-face in individual sessions or family sessions
Barnes and Summers (39) United Kingdom *N* = 2	Systematic and psychodynamic psychotherapy—couple with mild learning disability and cerebral palsy	Psychodynamic psychotherapy provided in practice has been shown to be beneficial for individuals with learning disabilities. Integration of psychodynamic and systematic ways appears to be useful when working with individuals with disabilities.	Not specified	Initial sessions were dedicated to learning about clients. The assessment period included clients drawing out genograms (pictorial way of representing family relationships) to determine significant events.Malan's triangles were used to create links between past and current perceptions of others.	Therapist on educational placement	Initially delivered through individual face-to-face sessions, followed by joint couple therapy sessions
Brown et al. (40) Australia *N* = 59	Stepping stones triple P (SSTP) and acceptance and commitment therapy (ACT)	Pediatric acquired brain injury can impact a child's cognitive, behavioral, emotional, ans social outcomes. The SSTP is a behavioral family intervention to prevent child emotional difficulties. ACT has been shown to enhance parent and child emotional outcomes. By combining both, there is a potential to see improved emotional and behavioral outcomes.	Not specified	Intervention consisted of 2-sessions of ACT and 9-sessions of SSTP. Specific intervention procedures were not specified.	Clinical psychologists or provisionally registered psychologist completing postgraduate training in clinical psychology; all had accreditation in SSTP	Delivered via face-to-face group therapy sessions, in groups size ranging from three to six families, and individual telephone sessions
Florou et al. (41) Greece *N* = 1	Psychoanalytic psychotherapy	Individuals born with physical disabilities may have difficulty with the integration of their body and self-image. This can consequently hinder the child's ability to form their identity, leading to increased mental health challenges. Short-term psychoanalytic psychotherapy may provide a treatment method to address the subsequent mental impact of youth's physical disability.	Not specified	Early sessions of therapy were dedicated to developing a positive working alliance between the therapist and client, by discussing his feelings toward his disability and his strengths/weaknesses. During the next phase of therapy, the client was encouraged to discuss his own behavior. The story of the Phantom of the Opera was used to help the client consciously work through his physical disability.	Therapist—nothing further specified in regard to title, training, and experience.	Delivered face-to-face with supervision (child's parents)
Golinska and Bidzan (42) Poland *N* = 1	Neuropsychological rehabilitation	Following a stroke, individuals may experience difficulties in cognitive function, emotional functioning, and coping with stress. The aims of neuropsychological rehabilitation is to reduce the negative side effects, specifically related to cognitive functioning. Improvements in this area can lead to improved quality of life in patients.	Not specified	Elements of cognitive-behavioral therapy (CBT) were used: identification of automatic thoughts, replacing automatic thoughts with alternative ones and relaxation sessions; Home exercises were completed; Non psychological therapy including cognitive skills training focusing on memory, attention, creativity, concentration, verbal fluency, and abstract thinking, in addition to neurofeedback.	Psychologist—nothing further specified in regard to training, or experience.	Delivered individually face-to-face
McCarty et al. (35) United States *N* = 4	Collaborative care for persistent post-concussive symptoms	Sports-related concussion in children and adolescents are often accompanied by post concussive and co-occurring psychological symptoms. This study was designed to investigate whether CBT embedded within the collaborative care treatment model would reduce post-concussive, anxiety, and depression symptoms in adolescents.	Not specified	Intervention included cognitive behavioral therapy (CBT), care management, and psychopharmacological consultation. CBT focused on post-concussive depression and anxiety consisting of coping skills, relaxation strategies, sleep hygiene, and positive thinking.	CBT—delivered by one of five study therapists (four PhD level psychologists, and one licensed therapist);	Therapy delivered in person, face-to-face.
McNally et al. (33) United States *N* = 31	Brief cognitive behavioral intervention and cognitive behavioral therapy (CBT)	Pediatric concussion is associated with a range of physical, cognitive, and emotional symptoms. Research indicates that CBT may treat prolonged post-concussive symptoms. A multi-faceted approach including elements of education, activity management, relaxation, and cognitive restructuring, is hypothesized to be beneficial to reduce patient symptoms and improve quality of life.	Families were provided with information about the treatment approach.	Treatment sessions consisted of the following modules: (1) psychoeducation—providing information to families regarding concussions and recovery; (2) activity and sleep scheduling, and sleep hygiene training; (3) relaxation training—including breathing, muscle relaxation, and relaxing imagery; and (4) cognitive restructuring—helping patients to identify and replace maladaptive thoughts	Licensed clinical psychologist specializing in neuropsychology or by doctoral and postdoctoral-level neuropsychology trainees under supervision.	Treatment was either delivered individually face-to-face or by joint sessions with both parent and child.
Pastore et al. (43) Italy *N* = 40	Cognitive behavioral therapy (CBT)	Difficulties following traumatic brain injury include anxiety and depression, and social deficits. Previous studies have shown that CBT is effective with young tumor survivors. Therefore, the effectiveness of CBT in improving psychological and behavioral problems could be promising.	Not specified	CBT intervention consisted of positive, negative, contingent, and intermittent reinforcement; chaining; shaping; prompting; fading; modeling; and extinction. Cognitive meditation and behavioral interventions were used including the ABC model (Antecedent, Behavior, Consequences model). Intervention structure: observation of behaviors in different settings, use of the ABC model, followed by individualized intervention plans. Psychoeducational interventions were directed at parents.	Two therapists of the institute provided therapy—no further information was specified.	Treatment was delivered face-to-face individually
Peterman et al. (36) United States *N* = 1	Cognitive-behavioral therapy (CBT)	The identification and treatment of anxiety disorders can be difficult for youth with physical disabilities such as cerebral palsy. In order to address anxiety within this population, it is important to consider concerns about mobility, social involvement, and self-care. As such, adapted CBT could be an effective treatment.	Not Specified	Initial sessions were used to create a therapeutic alliance. Subsequent sessions were devoted to behavior changes. Sessions one to five followed a section of the Coping Cat program, with aspects of Mastery of Anxiety and Panic integrated. Aspects of psychoeducation were also presented. Sessions six and seven focused on skill building and construction of exposure hierarchy. Subsequent sessions were dedicated to exposure, while teaching aspects of CBT.	Therapist—no further information was specified.	Treatment was delivered in individual face-to-face sessions with the client and their mother. Over the phone sessions occurred with the client's mother between in-person sessions.
Sylvester (37) United States *N* = 17	Acceptance and commitment therapy (ACT)	For individuals who have an acquired brain injury (ABI), psychological impairments following the injury can lead to decreased participation in meaningful activities. ACT has been shown to improve experiential avoidance, which is a result of individuals' psychological impairment. This study investigates the use of ACT group therapy on late effects of pediatric ABI.	Not specified	Treatment sessions targeted avoidance of difficult thoughts, feelings, bodily sensations/perceptual experiences, and self-attributions related to brain injury. Topics of sessions included: assessment of treatment targets, goals and values; successful working/creative hopelessness; control is the problem/willingness; mindfulness/defusion; self-as-context; self-compassion, integration of model, and values; values and committed action plan.	Group therapist. No further information specified.	Treatment was delivered face-to-face in group therapy sessions; groups consisted of three to five people.
Whiting et al. (38) United Kingdom *N* = 2	Acceptance and commitment therapy (ACT)	Following traumatic brain injury (TBI), impairments can occur in physical, cognitive, behavioral, emotional, and/or psychological domains. ACT has been used to promote psychological flexibility, rather than focusing on symptom reduction. Examined the feasibility of ACT in individuals in psychological distress after severe traumatic brain injury.	Not specified	In sessions tasks included psychoeducation, discussion, experiential exercises and instructions for a home task. Session titles included: introduction and confronting the agenda, control is the problem, acceptance and defusion, the observing self, introduction of values, values and committed action, and relapse prevention.	Intervention therapist. No further information was specified.	Treatment was delivered in group face-to-face therapy, the group consisted of two participants

*The TIDieR framework table was adapted from Hoffmann et al. (32)*.

### Intervention Types

Commonly identified psychological interventions used with individuals with childhood onset ABI and CP were acceptance and commitment therapy (ACT) (34, 37, 38, 40), psychotherapy (39, 41), and CBT (33, 35, 36, 42, 43), in combination with components of family therapy (34), psychoeducation (33, 36, 43), and Stepping Stones Triple P (SSTP) (40). Studies reporting on ACT were limited to individuals with childhood onset ABI, while studies investigating the use of psychotherapy only included individuals with CP. In contrast, the studies evaluating CBT included individuals with both childhood onset ABI (33, 35, 42, 43) and CP (36). Intervention characteristics of each included study can be found in Tables 1, 2.

**Table 2:** Summary of psychological interventions using the TIDieR framework items seven through 12.

**Item study**	**7.Where**	**8. When and how much**	**9. Tailoring**	**10. Modifications**	**11. How well (planned)**	**12. How well (Actual)**
Ashish et al. (34) United States *N* = 1	Outpatient behavioral health clinic	11 sessions of acceptance and commitment therapy (ACT) and seven sessions of family therapy	Not specified	Not specified	Not specified	Not specified
Barnes and Summers (39) United Kingdom *N* = 2	Community learning disabilities services	12 sessions	Therapy was provided at the clients' appropriate cognitive level including adapting vocabulary.	Therapy initially planned to be individual however the clients' partner joined during the second session and beyond. Clients did not want therapy resulting in the therapist giving them space, directing them to come when ready—provided clients with some control. Clients' chairs faced each other to symbolize they were the agents of change. Once therapy sessions were completed, they continued therapy with the therapist's supervisor.	Not Specified	Clients completed all 12 therapy sessions.
Brown et al. (40) Australia *N* = 59	Interventions occurred across five sites including hospitals, universities, or community venues across south-east queensland, Australia.	16 h (two ACT sessions for parents and six SSTPs) of group therapy; three (1.5 h) individual SSTP sessions over the phone; All were provided over 10 weeks	Not specified	Not specified	Practitioners received a nationally coordinated system of training and accreditation. They completed session checklists to assess content delivered. Independent observers assessed video/audio-recordings of 31% of group sessions to determine content delivered. Patient attrition was determined by participation in sessions and completion of post-assessment and post-follow up questionnaires. In the case of missed group sessions, parents were offered make-up sessions via telephone or face-to-face.	Group session checklists indicated that 100% of content was covered, and there was 99% agreement with an independent observer of video-recorded sessions. During telephone calls, 99% of content was covered. Fifty two of the 59 original participants (88%) completed the post assessment questionnaires, indicating an adequate retention rate. Dropouts completed between two and six sessions.
Florou et al. (41) Greece *N* = 1	Children's hospital	After the initial diagnostic session, treatment was delivered once a week for 12 months.	Therapy sessions were partially guided by the client, as they were able to talk about what they needed to.	Not specified	Not specified	The participant completed all sessions.
Golinska and Bidzan (42) Poland *N* = 1	Not specified	Therapy delivered over 1 year, with meetings two times per week; length of session was not stated.	Oriented to the particular patient—considered patient's resources, potential, and deficit areas.	States therapeutic plan underwent slight adjustment during execution, however specific details of how was not specified.	Not specified	The participant completed all sessions.
McCarty et al. (35) United States *N* = 4	Sports medicine and rehabilitation medicine clinic at seattle children's medical center and sports concussion program at harborview medical center	Treatment length was determined by patient duration of treatment and was terminated upon symptom resolution or at the end of 6 months.; mean number of CBT sessions was eight (range from 0 to 12)	Length of treatment times was dependent on the duration of their symptoms; therefore, treatment length was tailored to the individual patient.	Not Specified	A sample size of 40 provided adequate power for treatment affect, however 49 were recruited in order to compensate for potential dropout	The study attained >98% follow-up of the participants at one, three, and 6 months; 25 patients randomly assigned to the intervention, 23 completed the full course of collaborative care treatment over 6 months
McNally et al. (33) United States *N* = 31	Department of pediatric psychology and neuropsychology at nationwide children's hospital	Patients were seen for two to five treatment sessions, 45–60 min in duration; length of treatment varied; treatment occurred weekly.	Length of treatment time depended on clinical needs. Treatment was flexible, depending on presenting difficulties and treatment goals. Specific session content was based on clinical judgement of needs and preferences.	Only one patient received two additional treatment sessions beyond the five-session concussion treatment due to the need for ongoing monitoring/treatment of self-harm thoughts.	Adherence was assessed by patient attendance in treatment sessions, as reported by therapists.	Five patients dropped out before the last treatment session. (83.9% attrition)
Pastore et al. (43) Italy *N* = 40	Eugenio medea scientific institute unit, in italy	Treatment lasted 4–8 months occurring two or three times weekly; sessions lasted 45–60 min; a weekly session for parents was also scheduled.	After behavioral observation, individualized intervention plans were developed for each patient. Therefore, each intervention was tailored to the client.	Not specified.	Recruitment, pathological scores, as well as inclusion and exclusion criteria were used to narrow down included participants. Patient allocation to clinical treatment group and control group.	28 patients received treatment (clinical group) and 12 patients did not receive treatment (control group). Fourteen patients received CBT combined with a pharmacological intervention, 14 patients received only CBT, five patients received only drug therapy and seven patients received no treatment at all.
Peterman et al. (36) United States *N* = 1	University clinic specializing in the treatment of child and adolescent anxiety disorders.	24 1-h weekly sessions; Session 20–23 were biweekly then once per month; and by session 24 all goals were met.	The CBT protocol was tailored for the client by using components from the coping cat and mastery of anxiety and panic: riding the wave. psychoeducational material was presented in a developmentally sensitive way to enhance learning, such as through child-friendly metaphors; use of play, visual aids, and concrete presentation of concepts. Therapist consulted with the client's mother parents to explore safe vs. unsafe situations for client when constructing the exposure hierarchy	Therapy protocol was modified after the first few sessions to emphasize intrinsic and extrinsic motivation and introducing rewards earlier in the program. Some exposures had to be modified given the participant's physical limitations Exposures were not explained until the client reached the task at hand, nor were coping skills practiced extensively in advance due to client's extensive anxiety. The client missed several sessions due to being sick so “check-ins” with the client's mother were conducted via phone and were substituted for in-session therapy.	Not specified	Client completed all sessions
Sylvester (37) United States *N* = 17	Psychological services at the sierra regional center in reno, nevada	Eight weeks, with 1 weekly session	Modifications included: slow, simple speech; multimodal presentation (oral, pictorial, and physical); repetition of concepts; frequent monitoring of client comprehension and retention of concepts; allowing additional time to identify treatment targets, including difficult situations, thoughts, and feelings; utilizing examples from clients' lives.	Some groups had to undergo modifications to facilitate engagement among group members. Those who did not readily participate were encouraged to do so. For participants who had difficulties with articulation—therapists summarized their points. Participants who listened and summarized to assess comprehension, were modeled for other group members in order to promote fuller participation of all group members.	Treatment protocol was developed with targets for each therapy session; participants completed an adherence measure following each session to ensure that the protocol was followed; adherence ratings were obtained from participants; recruitment of individuals at clinic, and inclusion criteria; randomization to a treatment group	Overall adherence to protocol was 0.91; 30 clients were recruited; one participant did not show up for initial sessions but joined sessions four and five; 18 individuals met the inclusion criteria; five individuals withdrew during the course of the program. Two withdrew prior to group start, three withdrew after attending one group, one participant died during the program prior to post-treatment and follow-up; of the participants remaining, two were unable to be assessed at follow-up due to inability to contact the care coordinator.
Whiting et al. (38) United Kingdom *N* = 2	Outpatient services of liverpool brain injury rehabilitation unit in Australia	Seven weekly sessions, with each session lasting 1.5 h. The seventh sessions occurred after a one-month break.	The length of the session aimed to be of an appropriate time in order for both participants to tolerate and maintain focus.	Weekly phone call and day-of text message reminders were required to compensate for memory deficits and poor organizational abilities.; a 4 week break in the intervention protocol was required as one participant was in a motor vehicle accident—intervention resumed as planned following 4-week break	Behavioral observation of participants' completion of outcome measures and their engagement in the intervention protocol occurred. Behavioral observation included—whether items were missed on outcome measures, time taken to complete measures, participant comments, and whether assistance was required to complete measures. Participant attendance rates were recorded. Engagement looked at their ability to attend to the program, their degree of interaction in the program and engagement in homework tasks.	Both participants maintained 100% attendance; completed outcome measures, with assistance.

*The TIDieR framework table was adapted from Hoffmann et al. (32)*.

#### ACT Intervention

ACT interventions focused on increasing awareness of symptoms of distress, accepting them, and noticing when one was trying to avoid the associated thoughts, feelings and/or sensations. Mindfulness and cognitive defusion were key techniques used. Interventions involved group therapy sessions (37, 38, 40), with one study involving individual sessions (34). Groups ranged from two to six people. ACT interventions were also used in combination with family therapy with parents and children with ABI (34), and in combination with Stepping Stones Triple P (SSTP), a parenting program aimed at preventing child behavioral and emotional difficulties (40). ACT interventions all occurred in person.

#### Psychotherapy Intervention

Psychotherapy interventions involved revisiting an individual's past experiences to determine how they may be affecting daily life in the present. Interventions also aimed to extract meaning and break down feelings associated with one's disability. Interventions were delivered during individual sessions with parental supervision (41), or during couple's therapy sessions (39). The two included studies occurred in person and utilized initial sessions to build a therapeutic relationship with the clients. For instance, Barnes and Summers (39) used the activity of drawing genograms to represent family relationships. Florou et al. (41) used systemic and psychodynamic approaches, which consider the problem to belong within a whole system (e.g., the person and all the people in his/her life), while also considering the client's feelings and wishes regarding relationships in his/her life.

#### CBT Intervention

CBT interventions involved cognitive restructuring by identifying automatic thoughts and replacing them with more positive thinking. Clients participated in activity scheduling, sleep scheduling, and relaxation techniques. CBT interventions were delivered face-to-face. Peterman et al. (36) conducted sessions with clients and their mothers, while McNally et al. (33) delivered CBT interventions individually or with both the client and his/her parents. Pastore et al. (43) engaged clients in CBT techniques such as cognitive meditation, positive and negative reinforcement, contingent reinforcement and shaping, while Peterman et al. (36) utilized CBT as exposure therapy. Aspects of psychoeducation were also integrated into sessions for both clients and their parents (33, 36, 43). Intervention settings included hospitals (35, 43) and university clinics (36).

### Intervention Duration and Frequency

Individual sessions ranged from 45 min to 1.5 h, with many studies not indicating session duration (33, 38–40, 43). The majority of sessions occurred weekly (33, 37, 38, 41); however, some were twice or three times a week (42, 43). In the study by Peterman et al. (36), the frequency of sessions were tapered, starting with weekly sessions and progressing toward monthly sessions. Whiting et al. (38) provided clients with a 1-month break before the last session for relapse prevention. Interventions typically ranged from seven to 12 weeks (36–38, 40). In the study conducted by Pastore et al. (43), treatment duration ranged from 4 to 8 months, as treatment length was determined based on the participants' individualized needs. The longest treatment duration lasted 1 year (41, 42).

### Intervention Provider

Individuals providing psychological interventions were labeled under the broad term of “therapist.” In the study by Barnes and Summers (39), the therapist was completing an educational placement under the supervision of a therapist. In three of the 11 studies, psychologists facilitated sessions (33, 40, 42), while McCarty et al. (35) had psychologists and licensed therapists facilitate sessions. McNally et al. (33) identified a licensed clinical psychologist specializing in neuropsychology, or doctoral and postdoctoral-level neuropsychology “trainees” under supervision providing interventions. In terms of additional training to therapists providing interventions, one study indicated that training was provided in order to conduct SSTP (40).

### Intervention Effectiveness/Impact

The majority of ACT, CBT, and psychotherapy interventions were reported to be effective for the treatment of mental health symptoms in individuals with CP and ABI. Intervention outcome measures and findings can be found in Table 3.

**Table 3:** Summary of included studies' (*n* = 11) outcome measures and findings.

**Included study**	**Condition**	**Mean age in years (SD); age range years (SD)**	**Outcome measures**	**Findings**
Ashish et al. (34) United States *N* = 1	ABI—mTBI	14	Mini-mood and anxiety symptom questionnaire; therapist created 10-point “self-rating” scales to measure post-concussion symptoms; qualitative parent report	Self-rated anxiety reduced; client reported better attention, decreased fatigue, no difficulty with balance or speech, and higher trust in his physical and mental abilities; Patient reported reduced general distress, anxious arousal, and anhedonic depression; psychological symptoms improved; parent report stated client returned to baseline
Barnes and Summers (39) United Kingdom *N* = 2	Cerebral palsy	Not stated	Subjective report	Client able to talk about true hidden feelings; understanding of the problems improved; therapist reported evident that clients started to benefit from therapy
Brown et al. (40) Australia *N* = 59	ABI—varying causes	Intervention 7.13 (3.17), control 6.87 (3.03); not specified	Eyberg child behavior inventory; the strengths and difficulties questionnaire—emotional symptoms subscale; the parenting style	Short-term intervention effects on outcome measures; ACT and STTP group demonstrated significant improvements with treatment—decrease in behavior intensity and number, decrease in emotional symptoms; emotional scores returned to baseline at 6-month follow-up
Florou et al. (41) Greece *N* = 1	Cerebral palsy	15	Subjective report	Client was able to work through his disability and past trauma; client talked about anxieties and worries; therapist report it was difficult for the client to accept his disability, and the client managed to see himself differently; the body and mind became more unified leading to greater control over the client's body, as per therapist report
Golinska and Bidzan (42) Poland *N* = 1	ABI—TBI	15	Questionnaire for depression measurement; neuropsychological assessment	Severity of depressive symptoms decreased and mood in general improved; still significant fluctuations in mood; patient reported low or average levels of anxiety and psychosomatic symptoms; engagement level was found to influence therapy engagement level
McCarty et al. (35) United States *N* = 4	ABI—mTBI	Intervention 15.1 (1.6), Control 14.8 (1.7); 11–17	Patient health questionnaire (PQH-9); PROMIS-PA8 (version A); pediatric quality of life inventory—parent and youth report; client satisfaction questionnaire; health and behavior inventor	Clinically and significant improvements in postconcussive symptoms and health related quality of life in the treatment group; statistical improvements in health-related quality of life for the treatment group in child and parent report; greater reduction in depressive symptoms within treatment group compared to care as usual; treatment group had high levels of parent and patient satisfaction
McNally et al. (33) United States *N* = 31	ABI—mTBI	15.9 (2); not specified	Sport concussion assessment tool—third edition; pediatric quality of life inventory, v4.0; school attendance reported via self-report	Reduction in self-reported post-concussive symptoms over the course of treatment for all but one patient; all but one patient returned to full days of school after treatment; parent-reported quality of life significantly improved; significant improvement in quality of life domains, with the greatest magnitude of change in emotional and school functioning; success of treatment was not based on the length of time post injury
Pastore et al. (43) Italy *N* = 40	ABI—TBI	Interventions 10.91 (3.82), control 8.94 (3.32); not specified	Child behavior checklist (CBCL); the vineland adaptive behavior scales—expanded form	Significant advantage of several CBCL scales and a greater increase in adaptive behavior; treatment group showed greater decrease in behavioral and psychological problems, improved social skills, improved aggressive and externalizing behaviors
Peterman et al. (36) United States *N* = 1	Cerebral palsy	12 year old	Anxiety disorders interview schedule—child and parent versions; children's global assessment scale; clinical global impressions—severity and improvement; multidimensional anxiety scale for children—child and parent versions	Post-treatment, patient no longer met the criteria for an anxiety disorder, but continued to experience subclinical symptoms of separation anxiety and generalized anxiety disorder; decreased levels of anxiety
Sylvester (37) United States *N* = 17	ABI	Not specified; 12–59 years	Participation objective, participation subjective; mayo-portland adaptability inventory-fourth edition; orientation toward productive activities scale; symptom checlist-90-revised; avoidance and fusion questionnaire—youth; acceptance and action questionnaire-acquired brain injury; appraisal of threat and avoidance questionnaire; self compassion scale	Increased participation and decreased psychological distress following treatment; decreased functional disability, improving psychological health; decreased experiential and behavioral avoidance; participants reported greater participation in life activities
Whiting et al. (38) United Kingdom *N* = 2	ABI—TBI	19 and 29 years	Acceptance and action questionnaire—acquired brain injury; acceptance and action questionnaire-ii; hospital anxiety and depression scale; depression anxiety and stress scale-21; positive and negative affect scales; general health questionnaire-12; motivation for traumatic brain injury rehabilitation questionnaire; the sydney psychosocial reintegration scale-2; short form health survey	Patient one—gradual decrease in psychological distress and psychological inflexibility. Symptoms were still within the same clinical range. Patient two—significant decrease in psychological inflexibility and measures of mood. Significant increases in quality of life were reported.

*SD, standard deviation; TBI, traumatic brain injury; mTBI, mild traumatic brain injury; ABI, acquired brain injury*.

ACT interventions were reported to significantly decrease anxiety in individuals with post TBI mental health difficulties (34), decrease psychological distress in individuals with severe TBI (38), and decrease avoidance of thoughts and emotions, leading to greater life activity participation (37). When used with SSTP, ACT interventions were found to decrease problem behaviors, and significantly decrease emotional symptoms in individuals with ABI, compared to individuals receiving usual care (40).

Psychotherapy intervention impact was reported in terms of the therapists' opinions on patient progress, rather than the use of outcome measures (39, 41). For example, in the study by Florou et al. (41), the therapist stated psychotherapy was successful as the participant was better able to discuss anxieties and worries and process past traumas.

CBT interventions were effective in reducing depressive symptoms (35, 42, 43), improving emotional function as per parent reports (33), reducing anxiety levels (36, 42, 43), and decreasing behavioral and psychological problems (43). However, when investigating the maintenance of treatment effects post-intervention, mixed findings were reported. Individuals who received ACT and SSTP returned to baseline emotional symptoms after 6-months (40), while individuals receiving CBT continued to decrease in anxiety levels 1-month post-treatment (36).

In addition to the effectiveness of the interventions, two studies considered moderating/mediating variables of the main outcomes. For example, Golinska and Bidzan (42) considered participants' levels of engagement during interventions and its influence. With this, intervention success was affected by client engagement levels (42); with increased engagement associated with increased psychological resources and decreased depressive symptoms (42). Additionally, McNally et al. (33) examined whether length of time since ABI would influence participants' successful outcomes with a given intervention; however, it was found to have no effect.

## Discussion

The purpose of this scoping review was to: (1) determine what psychological interventions have been reported in the literature/evaluated to treat mental health difficulties experienced by individuals with CP, SB, and childhood onset ABI; and (2) identify the key characteristics of these interventions and their effectiveness. A total of 11 studies were included. The psychological interventions identified were ACT, CBT, and psychotherapy, in combination with family therapy, psychoeducation, and SSTP. No studies included individuals with SB. Included studies investigating childhood onset ABI mainly focused on concussions (e.g., mTBI), and TBI, which may limit the generalizability to all individuals with childhood onset ABI. The absence of studies on SB and a variety of ABI conditions, reveals the need for future research in these populations. The low yield of studies available for data abstraction highlights the need for further evidence and psychological support for these populations.

The most commonly reported psychological interventions leading to improved psychological outcomes, included CBT, ACT, and psychotherapy. Included studies provided evidence that CBT interventions decreased behavioral and psychological problems in children and adolescents with mild to moderate TBI (33, 35, 43), stroke (42), and CP (36). These results are similar to other literature investigating the effectiveness of CBT for reducing mental health symptoms amongst individuals with autism spectrum disorder (ASD) (44–46) and amongst individuals with epilepsy (47, 48). In a meta-analysis by Perihan et al. (44), findings from 23 studies suggested CBT interventions produced moderate changes in anxiety levels in children with ASD. Additionally, in a study by Carbone et al. (47), adolescents with epilepsy reported improved mental health and social functioning after completing a six module-based CBT intervention.

ACT intervention protocols were shown to improve mood and psychological outcomes in children and adults with childhood onset mild to severe TBI (34, 37, 38) and pediatric ABI (40). These findings are similar to results reported in a study by Pahnke et al. (49), looking at ACT-based skills training groups for adolescents and young adults with high-functioning ASD. The 6-week intervention was aimed at teaching participants acceptance and mindfulness skills to better deal with difficult thoughts, emotions, and bodily sensations (49). Positive outcomes were reported from both self- and teacher-reports in regard to decreased stress, hyperactivity, prosocial behavior and emotional symptoms (49).

Lastly, psychotherapy interventions were subjectively reported to improve mental health outcomes in individuals with CP (39, 41). Similar findings were found in individuals with ASD receiving psychotherapy interventions (50, 51). In a study by El-Ghoroury and Krackow (51), psychotherapy interventions resulted in decreased emotional outbursts with a client with ASD.

The findings from this review revealed heterogeneity in terms of key characteristics of intervention protocols across studies. Therefore, it is difficult to determine which intervention characteristics are associated with improved outcomes. Furthermore, some items of the TIDieR framework were also not described in the included studies, making it difficult to comprehensively describe the psychological interventions.

For many of the included studies, study protocols were tailored to the particular client in regard to length of treatment (33, 35), aspects of the intervention the client required (e.g., individualized treatment plans) (41, 43), and how material was presented (39). For example, the Modular Approach to Therapy for children with Anxiety, Depression, Trauma and Conduct problems (MATCH-ADTC), uses individualized treatment through the use of treatment modules (52). Modules can be repeated, or additional modules can be added, depending on the patient's response to treatment. This flexible and tailored treatment intervention has been reported to outperform usual care in youth with depression, anxiety, and conduct problems (53). Personalized interventions should utilize evidence-based methods to successfully tailor mental health treatment to clients (54). Evidence-based methods facilitate individualized mental health treatment planning and tailoring by assisting clinicians in determining what order to target problems, and what treatments to combine (53, 54). However, even when utilizing personalized interventions, not all intervention protocols may be feasible or beneficial for all clients with CP, SB, or childhood onset ABI. Future studies should seek to determine and understand how and why interventions are beneficial to certain individuals (54).

We acknowledge several strengths of this scoping review. This review benefitted from an extensive literature search conducted by an experienced informational specialist. The screening and extraction process were completed by two researchers independently. This review was also guided by two well-known frameworks, including PRISMA and TIDieR, ensuring both quality and transparency of studies included.

It is also important to acknowledge the limitations of this review. First, the literature search was limited to the last 10 years (e.g., 2009–2019), potentially excluding relevant studies published prior to 2009. Second, non-English studies were excluded, potentially creating a bias for English-speaking countries. Third, studies were excluded if they reported on non-traditional interventions such as music therapy or art therapy. Therefore, included studies may not encompass all interventions available. Fourth, the review is limited by a small number of available publications. Many articles did not include participant ages, therefore relevant studies may have been excluded. Relatedly, we included studies involving any participants age 12 and older (i.e., provided the studies met the other inclusion criteria). This meant including some studies with much younger children (e.g., 43, 46). While our original intent was to only include those studies with ≥50% of the sample age 12 and older, upon reviewing these studies, it was not possible to determine the proportion of the sample over the age of 12. Thus, we included these studies as a conservative measure given the relatively low yield of included studies for data abstraction. In the case of the studies involving psychotherapy interventions, impact was reported in terms of the therapists' opinions on patient progress, rather than the use of outcome measures. Lastly, the majority of the studies included small sample sizes, primarily being case studies. Case studies can be considered one of the least rigorous designs, with limited generalizability (55).

## Conclusions

This scoping review aimed to synthesize information regarding psychological interventions being evaluated for individuals with childhood onset ABI, CP and SB. CBT, psychotherapy and ACT were found to be effective interventions by decreasing mental health symptoms. Upon completing CBT, psychotherapy, or ACT, individuals with CP and childhood onset ABI, mainly TBI, experienced decreased anxiety, depressive symptoms, and psychological distress. The lack of literature pertaining to interventions for individuals with SB and different types of ABIs (e.g., tumors), in particular, highlights a need for future research within these populations. With modifications and personalization for these individuals, psychological intervention/treatment hold the potential to improve mental health outcomes and transitional care services.

## Author Contributions

LP ran the search strategy in the various databases. MJ and TP screened articles and abstracted data. All authors contributed equally to this work. All authors developed the search strategy and read and approved the final manuscript.

## Conflict of Interest

The authors declare that the research was conducted in the absence of any commercial or financial relationships that could be construed as a potential conflict of interest.

## Publisher's Note

All claims expressed in this article are solely those of the authors and do not necessarily represent those of their affiliated organizations, or those of the publisher, the editors and the reviewers. Any product that may be evaluated in this article, or claim that may be made by its manufacturer, is not guaranteed or endorsed by the publisher.
