# Supplementary material for: Psychological Interventions for Individuals With Acquired Brain Injury, Cerebral Palsy, and Spina Bifida: A Scoping Review
Source: Front Pediatr. 2022 Mar 21;10:782104. doi: 10.3389/fped.2022.782104 (PMC8978581; doi:10.3389/fped.2022.782104)
Supplement: Supplementary file 1 [file Data_Sheet_1.doc]

Supplementary File 1.

MEDLINE Search Strategy.

Database: Ovid MEDLINE: Epub Ahead of Print, In-Process & Other Non-Indexed Citations, Ovid MEDLINE® Daily and Ovid MEDLINE® <1946-Present>

Search Strategy:

--------------------------------------------------------------------------------

1 Cerebral Palsy/ [ childhood onset disabilities ]

2 (cerebral adj pals$).mp.

3 (little$ adj disease).mp.

4 (hemiplegi$ adj5 spastic$).mp.

5 (dipleg$ adj5 spastic$).mp.

6 (quadriplegi$ adj5 spastic$).mp.

7 (unilateral$ adj5 spastic$).mp.

8 (hemiplegi$ adj3 ataxi$).mp.

9 (dipleg$ adj3 ataxi$).mp.

10 (quadriplegi$ adj3 ataxi$).mp.

11 (unilateral$ adj3 ataxi$).mp.

12 exp Spinal Dysraphism/

13 (spina adj bifida).mp.

14 (spinal adj bifida).mp.

15 Meningomyelocele/

16 Meningocele/

17 myelomeningocele.mp.

18 meningocele.mp.

19 lipomyelomeningocele.mp.

20 (spinal adj dysraph$).mp.

21 Brain Injuries/

22 Craniocerebral Trauma/

23 exp Brain Hemorrhage, traumatic/

24 Brain Injuries, Diffuse/

25 exp Brain Injuries, Traumatic/

26 Brain Injury, Chronic/

27 Epilepsy, Post-Traumatic/

28 exp Stroke/

29 exp Hypoxia, Brain/

30 Brain Damage, Chronic/

31 Hematoma, Subdural, Intracranial/

32 Hematoma, Epidural, Cranial/

33 exp Meningitis/

34 exp Encephalitis/

35 apoplexy.mp.

36 concus$.mp.

37 encephalitis.mp.

38 meningitis.mp.

39 (cerebrovascular adj accident$).mp.

40 (brain adj2 injur$).mp.

41 (head adj2 injur$).mp.

42 (intracran$ adj2 injur$).mp.

43 (cerebr$ adj2 injur$).mp.

44 (cerebellar adj2 injur$).mp.

45 (brainstem adj2 injur$).mp.

46 (crani$ adj2 injur$).mp.

47 (skull$ adj2 injur$).mp.

48 (hemisphere adj2 injur$).mp.

49 (orbit$ adj2 injur$).mp.

50 (brain adj2 isch?em$).mp.

51 (head adj2 isch?em$).mp.

52 (intracran$ adj2 isch?em$).mp.

53 (cerebr$ adj2 isch?em$).mp.

54 (cerebellar adj2 isch?em$).mp.

55 (brainstem adj2 isch?em$).mp.

56 (crani$ adj2 isch?em$).mp.

57 (skull$ adj2 isch?em$).mp.

58 (hemisphere adj2 isch?em$).mp.

59 (orbit$ adj2 isch?em$).mp.

60 (brain adj2 infarc$).mp.

61 (head adj2 infarc$).mp.

62 (intracran$ adj2 infarc$).mp.

63 (cerebr$ adj2 infarc$).mp.

64 (cerebellar adj2 infarc$).mp.

65 (brainstem adj2 infarc$).mp.

66 (crani$ adj2 infarc$).mp.

67 (skull$ adj2 infarc$).mp.

68 (hemisphere adj2 infarc$).mp.

69 (orbit$ adj2 infarc$).mp.

70 (brain adj2 thrombo$).mp.

71 (head adj2 thrombo$).mp.

72 (intracran$ adj2 thrombo$).mp.

73 (cerebr$ adj2 thrombo$).mp.

74 (cerebellar adj2 thrombo$).mp.

75 (brainstem adj2 thrombo$).mp.

76 (crani$ adj2 thrombo$).mp.

77 (skull$ adj2 thrombo$).mp.

78 (hemisphere adj2 thrombo$).mp.

79 (orbit$ adj2 thrombo$).mp.

80 (brain adj2 emboli$).mp.

81 (head adj2 emboli$).mp.

82 (intracran$ adj2 emboli$).mp.

83 (cerebr$ adj2 emboli$).mp.

84 (cerebellar adj2 emboli$).mp.

85 (brainstem adj2 emboli$).mp.

86 (crani$ adj2 emboli$).mp.

87 (skull$ adj2 emboli$).mp.

88 (hemisphere adj2 emboli$).mp.

89 (orbit$ adj2 emboli$).mp.

90 (brain adj2 h?emorrhag$).mp.

91 (head adj2 h?emorrhag$).mp.

92 (intracran$ adj2 h?emorrhag$).mp.

93 (cerebr$ adj2 h?emorrhag$).mp.

94 (cerebellar adj2 h?emorrhag$).mp.

95 (brainstem adj2 h?emorrhag$).mp.

96 (crani$ adj2 h?emorrhag$).mp.

97 (skull$ adj2 h?emorrhag$).mp.

98 (hemisphere adj2 h?emorrhag$).mp.

99 (orbit$ adj2 h?emorrhag$).mp.

100 (brain adj2 h?ematoma$).mp.

101 (head adj2 h?ematoma$).mp.

102 (intracran$ adj2 h?ematoma$).mp.

103 (cerebr$ adj2 h?ematoma$).mp.

104 (cerebellar adj2 h?ematoma$).mp.

105 (brainstem adj2 h?ematoma$).mp.

106 (crani$ adj2 h?ematoma$).mp.

107 (skull$ adj2 h?ematoma$).mp.

108 (hemisphere adj2 h?ematoma$).mp.

109 (orbit$ adj2 h?ematoma$).mp.

110 (brain adj2 aneurysm$).mp.

111 (head adj2 aneurysm$).mp.

112 (intracran$ adj2 aneurysm$).mp.

113 (cerebr$ adj2 aneurysm$).mp.

114 (cerebellar adj2 aneurysm$).mp.

115 (brainstem adj2 aneurysm$).mp.

116 (crani$ adj2 aneurysm$).mp.

117 (hemisphere adj2 aneurysm$).mp.

118 (orbit$ adj2 aneurysm$).mp.

119 (brain adj2 hypoxi$).mp.

120 (head adj2 hypoxi$).mp.

121 (intracran$ adj2 hypoxi$).mp.

122 (cerebr$ adj2 hypoxi$).mp.

123 (cerebellar adj2 hypoxi$).mp.

124 (brainstem adj2 hypoxi$).mp.

125 (crani$ adj2 hypoxi$).mp.

126 (skull$ adj2 hypoxi$).mp.

127 (hemisphere adj2 hypoxi$).mp.

128 (orbit$ adj2 hypoxi$).mp.

129 (brain adj2 trauma$).mp.

130 (head adj2 trauma$).mp.

131 (intracran$ adj2 trauma$).mp.

132 (cerebr$ adj2 trauma$).mp.

133 (cerebellar adj2 trauma$).mp.

134 (brainstem adj2 trauma$).mp.

135 (crani$ adj2 trauma$).mp.

136 (skull$ adj2 trauma$).mp.

137 (hemisphere adj2 trauma$).mp.

138 (orbit$ adj2 trauma$).mp.

139 (brain adj2 lesion$).mp.

140 (head adj2 lesion$).mp.

141 (intracran$ adj2 lesion$).mp.

142 (cerebr$ adj2 lesion$).mp.

143 (cerebellar adj2 lesion$).mp.

144 (brainstem adj2 lesion$).mp.

145 (crani$ adj2 lesion$).mp.

146 (skull$ adj2 lesion$).mp.

147 (hemisphere adj2 lesion$).mp.

148 (orbit$ adj2 lesion$).mp.

149 (brain adj2 damage$).mp.

150 (head adj2 damage$).mp.

151 (intracran$ adj2 damage$).mp.

152 (cerebr$ adj2 damage$).mp.

153 (cerebellar adj2 damage$).mp.

154 (brainstem adj2 damage$).mp.

155 (crani$ adj2 damage$).mp.

156 (skull$ adj2 damage$).mp.

157 (hemisphere adj2 damage$).mp.

158 (orbit$ adj2 damage$).mp.

159 (brain adj2 oedema$).mp.

160 (head adj2 oedema$).mp.

161 (intracran$ adj2 oedema$).mp.

162 (cerebr$ adj2 oedema$).mp.

163 (cerebellar adj2 oedema$).mp.

164 (brainstem adj2 oedema$).mp.

165 (crani$ adj2 oedema$).mp.

166 (skull$ adj2 oedema$).mp.

167 (hemisphere adj2 oedema$).mp.

168 (orbit$ adj2 oedema$).mp.

169 (brain adj2 edema$).mp.

170 (head adj2 edema$).mp.

171 (intracran$ adj2 edema$).mp.

172 (cerebr$ adj2 edema$).mp.

173 (cerebellar adj2 edema$).mp.

174 (brainstem adj2 edema$).mp.

175 (crani$ adj2 edema$).mp.

176 (skull$ adj2 edema$).mp. (

177 (hemisphere adj2 edema$).mp.

178 (orbit$ adj2 edema$).mp.

179 (brain adj2 fracture$).mp.

180 (head adj2 fracture$).mp.

181 (intracran$ adj2 fracture$).mp.

182 (cerebr$ adj2 fracture$).mp.

183 (cerebellar adj2 fracture$).mp.

184 (brainstem adj2 fracture$).mp.

185 (skull$ adj2 fracture$).mp.

186 (brain adj2 contusion$).mp.

187 (head adj2 contusion$).mp.

188 (intracran$ adj2 contusion$).mp.

189 (cerebr$ adj2 contusion$).mp.

190 (cerebellar adj2 contusion$).mp.

191 (brainstem adj2 contusion$).mp.

192 (crani$ adj2 contusion$).mp.

193 (hemisphere adj2 contusion$).mp.

194 (orbit$ adj2 contusion$).mp.

195 (brain adj2 pressur$).mp.

196 (head adj2 pressur$).mp.

197 (intracran$ adj2 pressur$).mp.

198 (cerebr$ adj2 pressur$).mp.

199 (cerebellar adj2 pressur$).mp.

200 (brainstem adj2 pressur$).mp.

201 (crani$ adj2 pressur$).mp.

202 (hemisphere adj2 pressur$).mp.

203 (orbit$ adj2 pressur$).mp.

204 or/1-203

205 exp Behavior Therapy/

206 exp Psychotherapy/

207 Problem Solving/

208 Family Therapy/

209 Psychotherapy, Psychodynamic/

210 mindfulness.tw.

211 mbct.tw.

212 mbsr.tw.

213 "cognitive behavio?ral therap$".tw.

214 "cognitive behavio?ral intervention$".tw.

215 "cognitive behavio?ral treatment$".tw.

216 cbt.tw.

217 "stepping stones triple p".tw.

218 "acceptance commitment therap$".tw.

219 "behavio?ral parent train$".tw.

220 psychoeducation$.tw.

221 psycho-education$.tw.

222 (psychological adj therap$).tw.

223 psychotherap$.tw.

224 psycho-therap$.tw.

225 (mental adj health).tw.

226 (problem adj solving).tw.

227 (cognitive adj intervention$).tw.

228 (cognitive adj treatment$).tw.

229 (cognitive adj therap$).tw.

230 (family adj intervention$).tw.

231 (family adj treatment$).tw.

232 (family adj therap$).tw.

233 (behavio?r adj intervention$).tw.

234 (behavio?r adj treatment$).tw.

235 (behavio?r adj therap$).tw.

236 (psychological adj intervention$).tw.

237 (psychological adj treatment$).tw.

238 (relaxation adj therap$).tw.

239 (psychodynamic adj therap$).tw.

240 (psycho-dynamic adj therap$).tw.

241 (meta-cognitive adj therap$).tw.

242 (metacognitive adj therap$).tw.

243 or/205-242

244 204 and 243

245 exp Animals/ not (exp Animals/ and Humans/)

246 244 not 245

247 limit 246 to (english language and yr="2009 - 2019")
